# Synergistic Effects of Epoxidized Soybean Oil and Polyester Fiber on Crumb Rubber Modified Asphalt Using Response Surface Methodology

**DOI:** 10.3390/ma16093469

**Published:** 2023-04-29

**Authors:** Jie Pan, Jiao Jin, Shuai Liu, Mengcheng Xiao, Guoping Qian, Zhuo Wang

**Affiliations:** 1 School of Traffic and Transportation Engineering, Changsha University of Science & Technology, Changsha 410114, China; 202027010307@stu.csust.edu.cn (J.P.); shuailiu@stu.csust.edu.cn (S.L.); mengc0739@163.com (M.X.); guopingqian@csust.edu.cn (G.Q.); 2 International College of Engineering, Changsha University of Science & Technology, Changsha 410114, China; 202027010124@stu.csust.edu.cn

**Keywords:** crumb rubber, epoxidized soybean oil, polyester fiber, response surface methodology, storage stability

## Abstract

The incorporation of crumb rubber (CR) into asphalt pavement materials can improve the performance of asphalt pavement and generate environmental benefits. However, the storage stability of the crumb rubber asphalt (CRA) remains an issue that needs to be resolved. This study explores the interaction laws among various modified materials based on the response surface methodology. Optimal preparation dosages of each material are determined, and performance predictions and validations are conducted. The storage stability of the CRA compounded with epoxidized soybean oil (ESO) and polyester fiber (PF) is investigated by combining traditional compatibility testing methods with refined characterization methods. The results indicate that the modification of CRA exhibits better rheological properties when the percentages of CR, PF, and ESO are 22%, 0.34%, and 3.21%, respectively. The addition of ESO effectively complements the light components of CRA to improve asphalt compatibility, and the addition of PF alleviates the adverse effects of ESO’s softening effect on rheological properties through stabilization and three-dimensional strengthening. The scientifically compounded additions of ESO and PF can effectively enhance the storage stability and rheological properties of CRA, promoting the development of sustainable and durable roads.

## 1. Introduction

A substantial quantity of waste rubber tires and waste rubber materials are produced due to the fast growth of industry. This causes serious harm to the ecological environment and must be effectively treated [1]. Crumb rubber is a byproduct of the tire recycling process, made by grinding used tires into small particles [2]. Crumb rubber has good elasticity, resilience, and energy absorption, making it an ideal additive for modified asphalt [3]. The crumb rubber swells in the asphalt, and the cross-linking reaction between the effective components can form a mesh structure to improve the high- and low-temperature performances of asphalt [4]. Crumb rubber can also enhance asphalt’s elasticity and fatigue resistance, resulting in enhanced performance and a longer service life [5]. A high content (about 62% of the volume of modified asphalt) of crumb rubber can double the fatigue resistance of modified asphalt [6]. However, crumb rubber and asphalt are made from different materials and have different properties, which can make it difficult to achieve a homogeneous mixture [7]. A high content of crumb rubber can result in asphalt segregation and poor storage stability [8]. Therefore, it is necessary to improve the segregation and storage stability of crumb rubber asphalt while guaranteeing the dosage of crumb rubber in order to enhance the performance of asphalt pavement.

Different additives, such as compatibilizers, stabilizers, and viscosity reducers, have been used to solve this problem [9]. Chemical compatibilizers can improve the compatibility of crumb rubber with asphalt through the grafting reaction [10]. However, chemical compatibilizers have fewer practical applications owing to their toxicity. Viscosity reducers can improve the thermal storage stability of crumb rubber asphalt by breaking the long chains of rubber to form soluble radicals, or via grafting reaction [11]. Stabilizers exhibit a high dissolution with asphalt and form a stable cross-linked structure with enhanced compatibility with asphalt components [12]. Epoxidized soybean oil has been widely used as a vegetable oil-based plasticizer due to its excellent permeability, fluidity, non-toxicity, and non-hazardous nature [13]. The addition of epoxidized soybean oil supplements the asphalt’s light components and improves compatibility [14]. The epoxidized bonds in epoxidized soybean oil can react with the unsaturated bonds in asphalt via ring-opening to form compounds with a three-dimensional network structure [15], which improves the storage stability of the crumb rubber asphalt. The combination of ESO and Sasobit enhances the low-temperature performance of styrene–butadiene rubber modified asphalt (SBRMA), and mitigates the negative effects of short-term aging [16]. Although epoxidized soybean oil improves the thermal storage stability of crumb rubber asphalt, its softening effect deteriorates the asphalt’s high-temperature stability [17]. The stabilizing and reinforcing effect of fibers can compensate for this shortcoming.

Polyester fibers are synthetic fibers made from polyethylene terephthalate (PET) [18], with high strength, low weight, and a good resistance to UV radiation and chemical degradation [19]. Polyester fibers can improve the tensile strength, fatigue resistance, and crack resistance of asphalt [20]. The addition of polyester fiber leads to the formation of a fiber network structure within the asphalt mixture, enhancing its viscosity and high-temperature stability [21]. The mechanical properties of the asphalt concrete are optimal when the polyester fiber content is 1% [22]. The addition of polyester fiber can enhance the storage stability of the asphalt mixture while meeting the performance requirements of the pavement [23]. Therefore, the stabilizing, adsorbing, and reinforcing effects of polyester fiber have the potential to improve the compatibility of asphalt and it works in synergy with epoxidized soybean oil to improve the thermal stability of crumb rubber asphalt storage.

This study uses crumb rubber, polyester fiber, and epoxidized soybean oil as modified asphalt materials, which were modified by melting and mixing. BBR and MSCR experimental data were used as response indicators to establish response surface models to obtain scientific ratios of the three modified materials and to fully utilize the synergistic effects of the three materials. The rheological properties of modified asphalt at high and low temperatures were analyzed at the same time. Additionally, the softening point difference, fluorescence microscopy (FM), and scanning electron microscopy (SEM) were used to characterize the storage stability of the composite modified asphalt. This study contributes to the practical use of crumb rubber in asphalt binder to develop sustainable and durable pavements.

## 2. Materials and Methods

### 2.1. Materials

Polyester fiber: The basic technical indicators are shown in Table 1, purchased from Changzhou Tianyi Fiber Co., Ltd. (Changzhou, China). Epoxidized soybean oil: The basic technical indicators are shown in Table 2, purchased from Guangzhou Xinjinlong Plastic Additives Co., Ltd. (Guangzhou, China). Tire crumb rubber: 80 mesh (0.178 mm), purchased from Lantian Hongrui Industrial rubber dealership. Matrix asphalt: Basic technical performance indicators are shown in Table 3, purchased from Sinopec (Beijing, China).

### 2.2. Preparation of Composite Modified Asphalt

**Figure 1:**
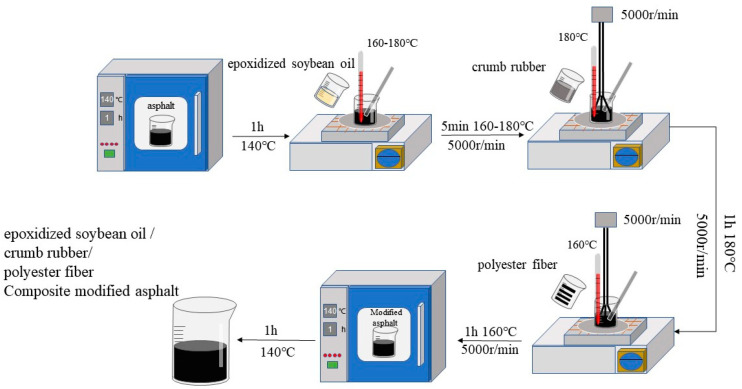
Preparation process of composite modified asphalt.

A preliminary investigation was carried out using a test study methodology [24,25] based on prior research, with the content of polyester fiber set between 0.2% and 0.4%, tire crumb rubber at 18% to 22%, and epoxidized soybean oil at 2% to 6%, all relative to the mass of the matrix asphalt. The preparation process parameters for 17 groups of composite modified asphalt were determined using the Box-Behnken design, as shown in Table 4. The matrix asphalt was heated to the liquid state and subjected to high-speed shearing at a rotational speed of 5000 r·min^−1^. The process involved mechanically stirring the epoxidized soybean oil at 160 °C to 180 °C for 5 min, followed by crumb rubber stirring at 180 ± 5 °C for 1 h, and polyester fiber stirring at 160 ± 5 °C for 1 h. The composite modified asphalt was finally prepared by swelling and developing in an oven at 140 °C for 1 h. The preparation process is illustrated in Figure 1. The modified asphalt is prepared via commercial melting and mixing. During the production period in the premix plant, it is sufficient to heat the asphalt binder to the appropriate temperature range and then to sequentially add the modified materials for adequate mixing so that the modified materials are uniformly dispersed in the asphalt. The production process is relatively simple and easy to implement.

### 2.3. Tests

**Figure 2:**
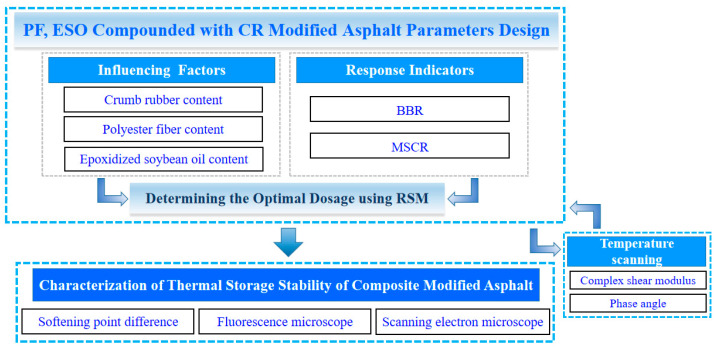
Flow chart of the experimental plan.

The relationship between the contents of modified materials and the experimental data of BBR and MSCR are established based on the response surface method. The effects of different modifiers on the rheological properties of asphalt are compared using temperature scanning. Finally, softening point difference, fluorescence microscopy (Leika DM4, Germany), and scanning electron microscopy (EVO10, Germany) are combined to characterize the storage stability of the composite modified asphalt. The flow chart of the experimental plan is shown in Figure 2.

#### 2.3.1. Rheological Properties of Asphalt

A dynamic shear rheometer (DSR) was utilized on the asphalt samples according to ASTM D7405-2010a [26] by using an Anton Paar MCR 301 device from Austria. A multiple stress creep recovery test (MSCR) was conducted, and its non-recoverable creep compliances at 0.1 kPa and 3.2 kPa were employed as evaluation indices of the high-temperature rheological properties of the asphalt [27]. The bending beam rheometer (BBR) test was used to evaluate the low-temperature rheological properties of composite modified asphalt in accordance with ASTM D6648-08(2016) [28] by using a TE-BBR device from America, with the creep rate (*m*) and stiffness modulus (*S*) at −18 °C for 60 s serving as evaluation indices. The asphalt samples were tested via temperature scanning (scanning range: 40–80 °C), and the phase angle (δ) and complex shear modulus (G*) were used as the evaluation indexes of high-temperature rheological properties.

#### 2.3.2. Storage Stability Tests

**Figure 3:**
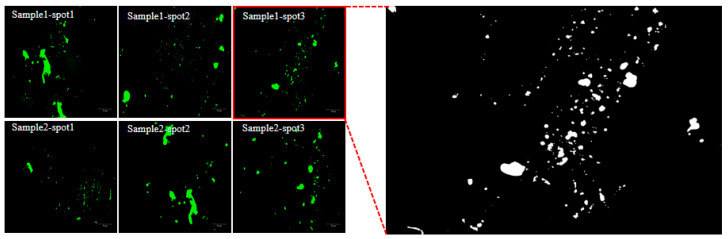
Six images of each part of modified asphalt (**left**) and quantitative analysis results (**right**).

The procedure for evaluating the storage stability of the composite modified asphalt involved pouring the sample into a 2.5 cm segregation tube, incubating it in a 163 ± 5 °C oven for 48 h, and then transferring it to −12 °C for 4 h, as specified in JTG E20-2011 [29]. The softening point difference was then measured at the upper and lower sections of the segregation tube to assess the stability of the asphalt [30]. Fluorescence microscopy (FM) was utilized to observe the upper and lower sections of the asphalt in the segregation tube after segregation with a magnification of 50 μm, to assess the degree of asphalt segregation based on the dispersion concentration of the modified substances [31]. Two samples were taken from each of the upper and lower sections of the separation tube, and three points were taken for each sample. This made a total of six images generated for each asphalt section to avoid statistically significant errors [32]. The quantification of the asphalt microstructure was carried out using Fiji ImageJ (NIH, Bethesda, Maryland, USA) by enabling the standard threshold selection to mark the green parts, as shown in Figure 3. The microscopic distribution characteristics of the three crumb rubber modified asphalts were further investigated using the stability coefficients. Scanning electron microscopy (SEM) was also used to observe the morphology of modified asphalt and to characterize its storage stability.

### 2.4. Response Surface Methodology

The study aims to examine the effect of the quantity of epoxidized soybean oil, crumb rubber, and polyester fiber on the high- and low-temperature performances of composite modified asphalt. Seventeen groups of composite modified asphalt were prepared using the Box-Behnken design with varying quantities of the three modifiers as a percentage of the matrix asphalt mass. The influences of the dosages of these modifiers on the performance of the composite modified asphalt were analyzed using regression analysis. It was necessary to adopt a systematic and appropriate method of data processing and analysis, in order to assure the validity of the results and to meet the desired performance requirements.

The Response Surface Methodology (RSM) is a statistical technique that combines the design of experiments and mathematical modeling to establish a second-order polynomial relationship between the design variables and the response variables, thereby determining the optimal combination of design variables and the corresponding optimal response value [33]. RSM has the advantage of high precision and reduced experimentation, which makes it suitable for the analysis of multi-factor interactions and the prediction of target values based on different levels of design variables. RSM has been extensively employed in the optimization of processes and enhancement of product parameters in pavement technology [34].

## 3. Results and Discussion

### 3.1. Response Surface Methodology

In this section, the optimization and analysis of the properties of composite modified asphalt composed of polyester fiber, crumb rubber, and epoxidized soybean oil were performed. The study utilized 17 groups of composite modified asphalt prepared based on the previously designed test scheme. The influencing factors considered in the study included the content of polyester fiber, crumb rubber, and epoxidized soybean oil. The creep rate, stiffness modulus, and non-recoverable creep compliance at 0.1 kPa and 3.2 kPa were used as response indicators to characterize the high- and low-temperature properties of the composite modified asphalt. Results from the experiments were used to establish the response surface model that predicts the optimal dosage of modified materials.

The degree of approximation of the response surface was evaluated using the complex correlation coefficient (*R*^2^) and correction determination coefficient (*Adj.R*^2^), as indicated by the calculation formulas in Equations (1) and (2). The results of the model’s variance analysis are shown in Table 5.

(1) $$R^{2}=\frac{{\mathrm{SS}}_{\mathrm{residual}}}{{\mathrm{SS}}_{\mathrm{model}}{+\mathrm{SS}}_{\mathrm{residual}}}$$

(2) $$\mathrm{Adj}{.R}^{2}=1-\frac{\frac{{\mathrm{SS}}_{\mathrm{residual}}}{{\mathrm{DF}}_{\mathrm{residual}}}}{\frac{{\mathrm{SS}}_{\mathrm{model}}-{\;\mathrm{SS}}_{\mathrm{residual}}}{{\mathrm{DF}}_{\mathrm{model}}{+\mathrm{DF}}_{\mathrm{residual}}}}$$

In the equation above: *SS_residual_* is the residual sum of squares; *SS_model_* is the model sum of squares; *DF_residual_* is the residual degree of freedom; *DF_model_* is the model degree of freedom.

#### 3.1.1. Low-Temperature Rheological Property

**Figure 4:**
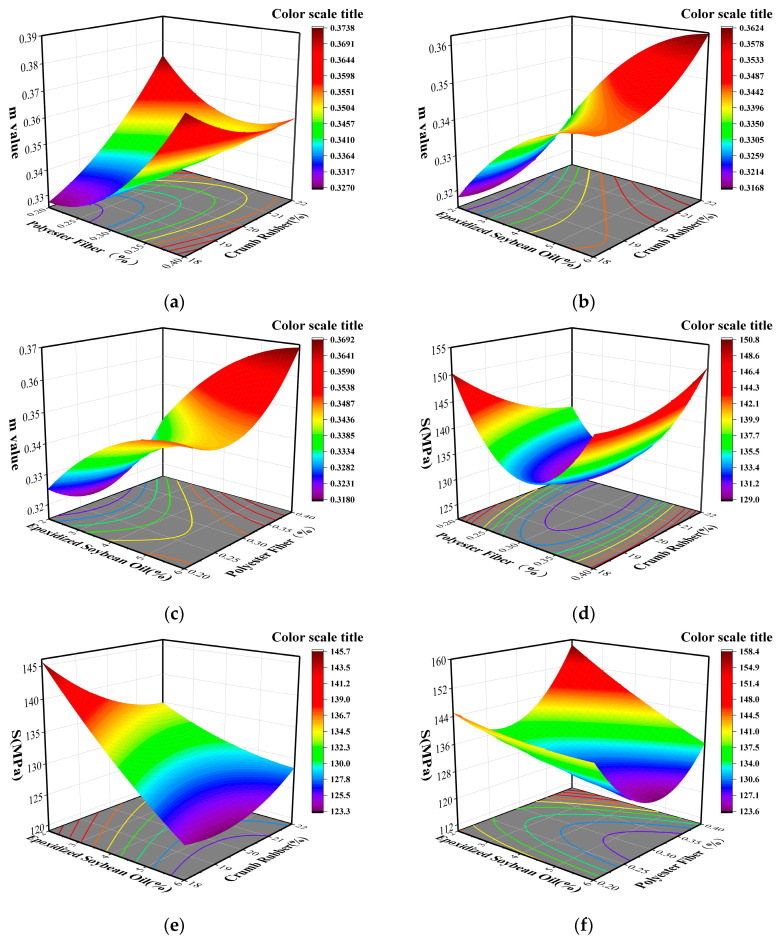
Interaction between various test factors and low-temperature index of composite modified asphalt. (**a**) Effect of interaction between CR and PF; (**b**) Effect of interaction between CR and ESO; (**c**) Effect of interaction between ESO and PF; (**d**) Effect of interaction between CR and PF; (**e**) Effect of interaction between CR and ESO; (**f**) Effect of interaction between ESO and PF.

The low-temperature creep characteristics of the composite modified asphalt were evaluated using the bending beam rheometer test (BBR). An increase in *m* and a decrease in *S* are indicative of an enhancement in the anti-cracking performance of the asphalt pavement at low temperatures. The response surface diagram was created using Design Expert software from Stat-Ease, Inc. (Minneapolis, Minnesota, USA), and the results show that when the polyester fiber content is kept constant, the increase in crumb rubber content results in an increase in *m* and a decrease in *S*, as depicted in Figure 4a,d. It indicates that the addition of crumb rubber improves the rheological properties of asphalt at low temperatures due to the cross-linking reaction of crumb rubber in asphalt, which increases the asphalt’s toughness at low temperatures. Figure 4a,d also shows that as the amount of polyester fiber increases, the *m* and *S* values initially decrease and then increase, while the amount of crumb rubber remains constant. Additionally, an increase in epoxidized soybean oil leads to a rise in *m* and a drop in *S* when the crumb rubber content is constant, as shown in Figure 4b,e, thereby enhancing the low-temperature crack resistance of the modified asphalt. This is due to the fact that the epoxidized soybean oil complements the lighter components of the asphalt. A comprehensive analysis of the results demonstrates that a suitable amount of polyester fiber can improve the low-temperature performance of the modified asphalt by forming a three-dimensional network structure within it [35]. The addition of epoxidized soybean oil can also enhance the rheological properties of asphalt at low temperatures.

The following coded multiple quadratic regression equation can be derived from the experimental results of the *m* and *S* values:

(3) $$\begin{array}{ll} m=0.4566- & 0.033625X+0.85775Y+0.013112Z\\ & \;-0.0775\mathrm{XY}+0.00025\mathrm{XZ}+0.00875\mathrm{YZ}+0.001494X^{2}+1.2225Y^{2}-0.001694Z^{2}\; \end{array}$$

(4) $$S=711.075-35.8125X-1173.25Y-17.525Z+22.5\mathrm{XY}+0.9375\mathrm{XZ}-23.75\mathrm{YZ}+0.6125X^{2}{+1395Y}^{2}+0.3Z^{2}$$

The response surface model was subjected to variance and significance analysis, with the results presented in Table 5. The *p* and *F* values indicate the significance of the correlation coefficient [36]. As seen in Table 5, the *p* values for the creep rate (*m*) and stiffness modulus (*S*) are 0.0003 and 0.0005, while the corresponding *F* values are 21.03 and 18.28, respectively. The *p* values are below the significance threshold of 0.05, indicating that the model exhibits significant correlation and statistical significance. The complex correlation coefficients (*R*^2^) of the creep rate and stiffness modulus are 0.96 and 0.95, and the adjusted determination coefficients (*Adj.R*^2^) are 0.91 and 0.90, respectively. The closer the values of *R*^2^ and *Adj.R*^2^ are to 1, the better the fit of the regression equation and the closer the correlation between the predicted and actual values. This model can be employed to analyze and to predict related indexes.

#### 3.1.2. High-Temperature Rheological Property

**Figure 5:**
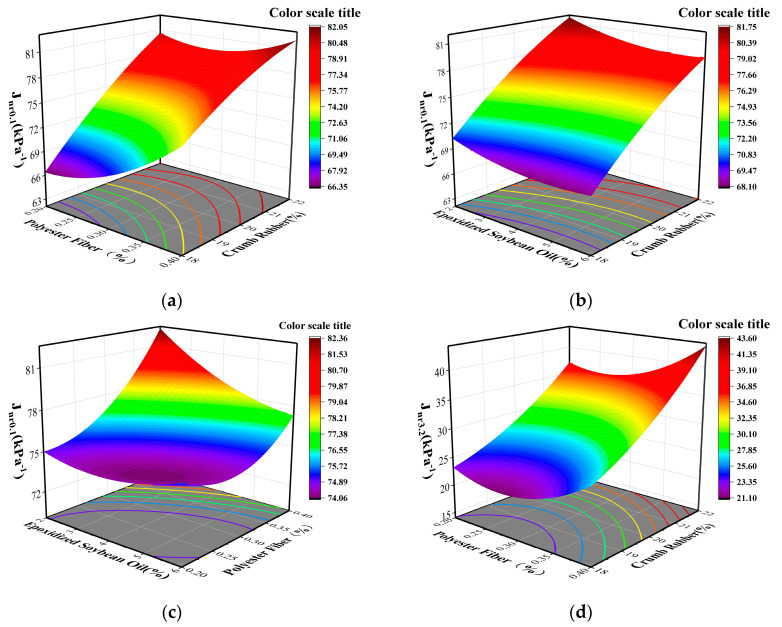

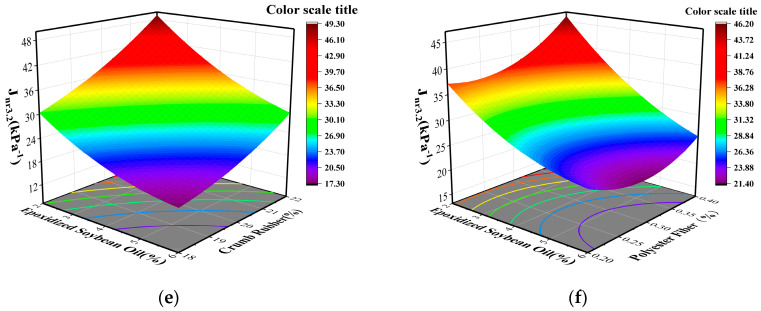
Interaction between various test factors and high-temperature index of composite modified asphalt. (**a**) Effect of interaction between CR and PF; (**b**) Effect of interaction between CR and ESO; (**c**) Effect of interaction between ESO and PF; (**d**) Effect of interaction between CR and PF; (**e**) Effect of interaction between CR and ESO; (**f**) Effect of interaction between ESO and PF.

A multiple stress creep recovery test (MSCR) was conducted on 17 groups of composite modified asphalt to determine their non-recoverable creep compliance at 0.1 kPa and 3.2 kPa. The utilization of Design Expert software enabled the creation of three-dimensional response surface diagrams. The analysis of the results depicted in Figure 5 suggests that an increase in the content of crumb rubber leads to a rise in the non-recoverable creep compliance and a decrease in the permanent deformation resistance of asphalt under varying levels of stress when the content of polyester fiber remains constant. On the other hand, the resistance to the permanent deformation of asphalt increases and then slows down with the increase in the amount of epoxidized soybean oil, as shown in Figure 5b,e, when the amount of crumb rubber is kept constant. Figure 5c,f show that the addition of polyester fiber mitigates the detrimental impact of the softening effect of epoxidized soybean oil on the high-temperature rheological properties of asphalt. The results further indicate that a suitable content of polyester fiber can boost the permanent deformation resistance of asphalt and improve the high-temperature properties of asphalt.

The following coding multiple quadratic regression equation can be derived from the experimental results:

(5) $$\begin{array}{ll} J_{\mathrm{nr}0.1}=-111.657 & +14.02687X+96.9325Y+0.79275Z-7.8375\mathrm{XY}-0.05375\mathrm{XZ}-7\mathrm{YZ}\\ & -0.217062X^{2}+184.175Y^{2}+0.227312Z^{2}\; \end{array}$$

(6) $$\begin{array}{ll} J_{\mathrm{nr}3.2}=151.8325 & -13.02375X-225.0123Y+2.78062Z+3.575\mathrm{XY}-0.445\mathrm{XZ}\\ & -9.8375\mathrm{YZ}+0.439063X^{2}+363.875Y^{2}+0.617188Z^{2}\; \end{array}$$

Variance analysis was performed on the multiple quadratic regression equation established based on the results of the multiple stress creep recovery test (MSCR) conducted on 17 groups of composite modified asphalt. The results of the analysis are presented in Table 5. It can be observed from Table 5 that the complex correlation coefficients (*R*^2^) for *J_nr_*_0.1_ and *J_nr_*_3.2_ are 0.96 and 0.98, respectively, and that the adjusted determination coefficients (*Adj.R*^2^) are 0.93 and 0.97. These values, which are close to 1, indicate a high degree of fitting and accuracy for the model, and this model can be utilized to predict the change behavior of *J_nr_*_0.1_ and *J_nr_*_3.2_.

#### 3.1.3. Design Optimization Comparison

**Figure 6:**
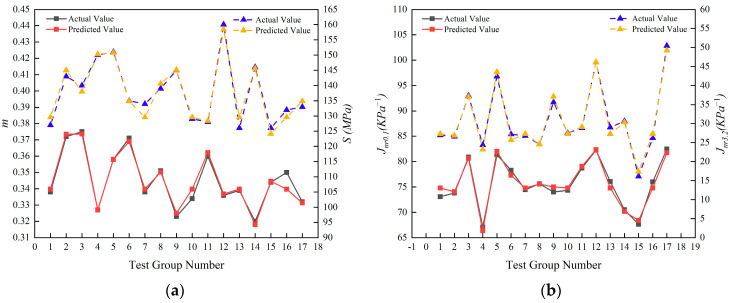
Comparison of predicted and actual values; (**a**) Comparison of measured creep rate and stiffness modulus with the predicted values of the model; (**b**) Comparison of measured non-recoverable creep compliance with the predicted values of the model.

A response surface model for each index was established based on the results above, and its predicted value was compared with the experimentally measured value, as illustrated in Figure 6a,b. A strong correlation is observed between the predicted and measured values, indicating that the model can be used effectively to analyze and to predict the relevant indicators.

Figure 6a,b demonstrates a high degree of concordance between the predicted values and the corresponding actual values. These results provide the basis for optimizing design variables to specific requirements and expectations in practical engineering applications. In this study, an optimization of the response surface model was performed to determine the optimal design parameters for the composite modified asphalt. The optimal composition was found to be 22% crumb rubber, 0.34% polyester fiber, and 3.21% epoxidized soybean oil, relative to the mass of the matrix asphalt. The composite modified asphalt exhibits the best overall performance under these conditions. Compared to a commercial asphalt binder such as SBS, the percentage of modified materials added in the above ratio is comparatively low, and the average market prices of crumb rubber, polyester fiber, and epoxidized soybean oil are not high. The current prices of polyester fiber, epoxidized soybean oil, and crumb rubber are relatively cheap. The price of SBS modified asphalt is USD 880 per ton, while the cost of composite modified asphalt is about USD 710 per ton according to the best addition ratio for SBS modified asphalt. Modified materials are less costly and more cost-effective. From a long-term perspective, this composite modified asphalt can extend the service life of asphalt pavement, reduce the number of asphalt pavement repairs, and lower pavement maintenance costs, with certain economic benefits and investment value.

### 3.2. Experimental Analysis of Temperature Scanning

**Figure 7:**
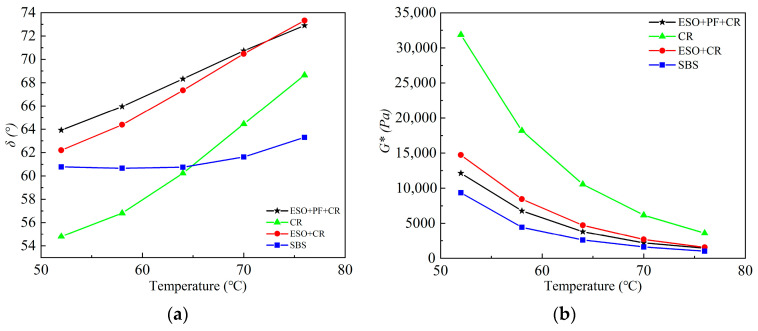
Temperature scan experimental data graph. (**a**) Phase angles of different modified asphalts; (**b**) Complex shear moduli of different modified asphalts.

Four modified asphalt phase angles (δ) and complex shear moduli (G*) of CR, ESO + CR, ESO + PF + CR, and SBS are compared and analyzed. SBS modified asphalt is chosen as the reference. Figure 7a shows that the phase angle of different modified asphalts changed positively with temperature, except for SBS modified asphalt. This symbolizes the asphalt’s increase in viscosity and decrease in elasticity. The particular variation in the phase angle of SBS modified asphalt may be attributed to the inherent nature of the SBS modifier itself. The phase angles of CR, ESO + CR, and ESO + PF + CR modified asphalt all increase with increasing temperature. The curve of CR modified asphalt is located below the curve of SBS modified asphalt at temperatures below 64 °C, indicating that its ability to resist deformation is higher than that of SBS modified asphalt at lower temperatures. It is worth noting that the curve of ESO + CR intersects with the curve of ESO + PF + CR at around 70 °C, and then the curve of ESO + PF + CR modified asphalt is lower than that of ESO + CR modified asphalt. This indicates that at higher temperatures, the reinforcing and stabilizing effect of PF mitigates the adverse effects of ESO softening on the high-temperature deformation resistance of asphalt, and the addition of PF improves the high-temperature rheological properties of modified asphalt. The complex shear modulus of the four modified asphalts falls monotonically as the temperature rises, as shown in Figure 7b, and the change trend is essentially the same. The complex shear moduli of CR, ESO + CR, and ESO + PF + CR modified asphalt are always higher than that of SBS modified asphalt, indicating that the addition of modifiers effectively improves the high-temperature rheological properties of asphalt.

### 3.3. Storage Stability

A partial three-dimensional network structure forms between crumb rubber and asphalt as the content of crumb rubber increases, leading to heightened segregation [25]. Despite the fact that crumb rubber asphalt improves the performance of asphalt, the compatibility issues between the two materials results in segregation during construction and transportation. Thus, it is important to find measures to stabilize rubber modified asphalt and to increase its usage. This section utilized the comparative analysis of asphalt storage stability via the softening point difference, fluorescence microscopy, and scanning electron microscopy to investigate the influence of polyester fiber (PF) and epoxidized soybean oil (ESO) on the storage stability of crumb rubber modified asphalt.

#### 3.3.1. Softening Point Difference after Segregation

The softening point difference, a commonly employed index for evaluating the storage stability of modified asphalt, was utilized in this study to examine the influence of PF and ESO on the storage stability of crumb rubber modified asphalt. The experiment involved storing various modified asphalt samples at 163 °C for 48 h and then dividing the sealed aluminum tubes into three sections. The upper and lower sections were selected for softening point analysis, with the difference in softening point between the sections being used to assess the degree of segregation and storage stability. The results are presented in Table 6.

The analysis of Table 6 reveals that the difference in the softening point between the upper and lower sections of the modified asphalts ESO + PF + CR, ESO + CR, and CR after segregation are less than 2.5 °C, fulfilling the specification requirements. The CR modified asphalt has the biggest softening point difference among the three modified asphalts, at 1.75 °C. The addition of ESO results in a reduction in the softening point difference of ESO + CR to 1.6 °C, likely due to the ring-opening reaction between the epoxidized bond in ESO and the unsaturated bond in asphalt, which forms a network structure compound and improves the compatibility between crumb rubber and asphalt [15]. The ESO + PF + CR modified asphalt has the lowest softening point difference at 0.85 °C, indicating minimum segregation and the best storage stability. The stability of ESO + PF + CR modified asphalt is greatly improved through the combination of the multi-directional reinforcement of polyester fiber [37] and the improved compatibility between crumb rubber and asphalt due to ESO. The softening point difference of ESO + PF + CR modified asphalt is reduced by 46.9% relative to ESO + CR, and by 51.9% relative to CR, demonstrating a considerable improvement in storage stability and a decrease in crumb rubber segregation within asphalt under the synergistic effects of ESO and PF.

#### 3.3.2. Fluorescence Microscope Test after Segregation

**Figure 8:**
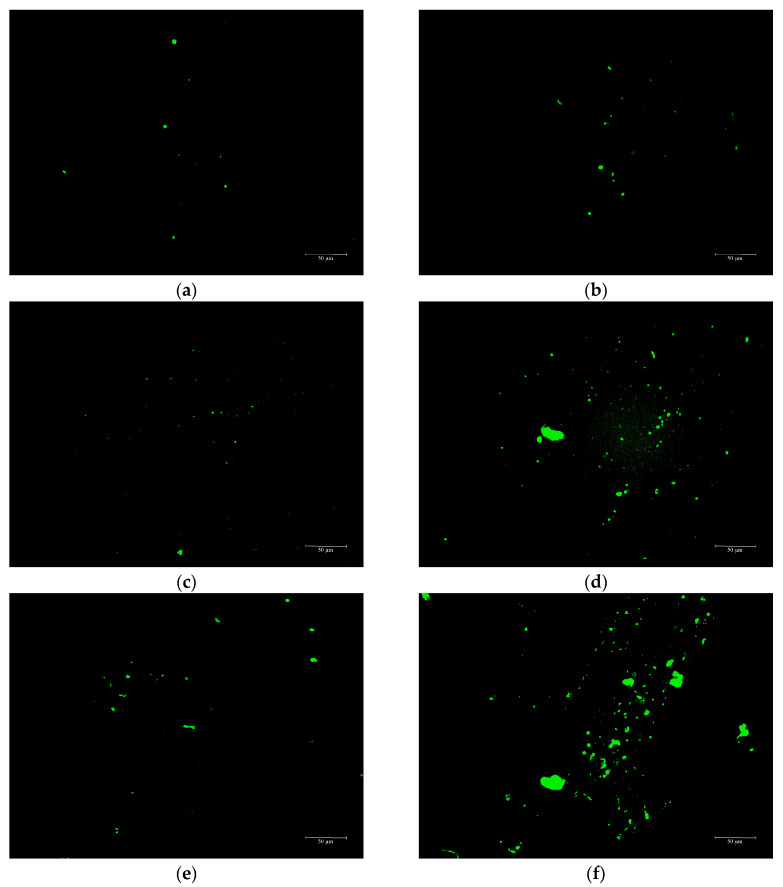
Fluorescence microscopy images of three modified asphalts after 48 h storage. (**a**) ESO + PF + CR upper sections; (**b**) ESO + PF + CR lower sections; (**c**) ESO + CR upper sections; (**d**) ESO + CR lower sections; (**e**) CR upper sections; (**f**) CR lower sections.

The use of fluorescence microscopy has been shown to be an effective tool for observing the phase structures of polymers in the process of blending polymers and asphalt and for providing insight into the morphological structure of modified asphalt. This technique works by exposing the polymer in the modified asphalt to light of a specific wavelength, which causes the energy absorbed by the polymer to jump to an excited state and emit fluorescence [38]. The fluorescence microscope was utilized to examine the modified materials dispersions of ESO + PF + CR, ESO + CR, and CR in the upper and lower sections of a segregation tube after being stored at 163 °C for 48 h. This investigation was performed to assess the thermal storage stability of asphalt, and the results are presented in Figure 8.

Figure 8 shows that in the comparison between the upper and lower sections of ESO + PF + CR, ESO + CR, and CR, the fluorescent substance difference in ESO + PF + CR is minimal, indicating a more uniform dispersion. This can be attributed to the stability provided by PF adsorption and three-dimensional reinforcement, as well as improved compatibility due to ESO. In contrast, the difference in fluorescent substances between the upper and lower sections of CR modified asphalt in Figure 8e,f is the largest, with an obvious accumulation of fluorescent substances in the lower section. ESO may have enhanced asphalt compatibility, promoting gradual fusion with the fluorescent material, which results in uniform dispersion and reduced segregation, according to Figure 8c,d. CR modified asphalt exhibits the highest amount of fluorescent substances based on the analysis of fluorescent substances in ESO + PF + CR, ESO + CR, and CR in Figure 8. This can be attributed to the incomplete decomposition and dispersion of rubber particles, which are then exposed using fluorescence microscopy. On the other hand, the fluorescent substance in ESO + CR is relatively lower than that of CR alone, due to the promoting effect of ESO on the compatibility between CR and asphalt. Moreover, ESO + PF + CR displays the best storage stability and the least amount of fluorescent substance due to the multi-directional reinforcement and stabilization provided by PF, and the improved compatibility of ESO with asphalt.

Fiji ImageJ was utilized to quantitatively assess the fluorescent area of the added substance in the asphalt binder. Additionally, the stability coefficient (*I_se_*) of the modified asphalt was computed using Formula (7). The closer the value of *I_se_* is to 1, the better the storage stability of the modified asphalt is. The results of this analysis are presented in Table 7.

(7) $$I_{\mathrm{se}}=\frac{S_{t}}{S_{b}}\;$$

In the equation above: *S_t_* and *S_b_* represent the fluorescence areas of the upper and lower sections of the segregation tube, respectively.

Table 7 shows that the fluorescent area of ESO + PF + CR composite modified asphalt is smaller compared to the other two types of modified asphalt. Therefore, the segregation of ESO + PF + CR composite modified asphalt is not obvious. The stability coefficient of ESO + PF + CR composite modified asphalt has an *I_se_* value that is approximately 2.1 times higher than that of CR asphalt and 1.2 times higher than that of ESO + CR asphalt, suggesting that it has a high level of stability. Furthermore, its stability coefficient’s *I_se_* value is closest to 1, indicating that the degradation degree of modified substances is high, with uniform dispersion and forming a stable system within the matrix asphalt, which is consistent with the previous analysis.

#### 3.3.3. Scanning Electron Microscopy Analysis

Scanning electron microscopy (SEM) was used to observe the microscopic morphology of the modified asphalt and to compare the compatibilities of different modified materials with asphalt. Then, the binding mechanism and storage stability of the modified material with asphalt were analyzed.

**Figure 9:**
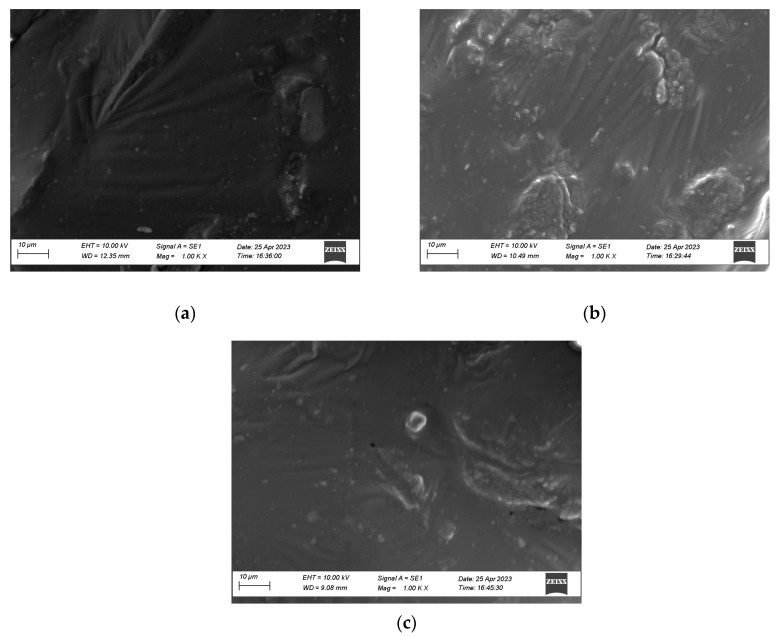
Scanning electron micrograph. (**a**) Matrix asphalt; (**b**) ESO + CR; (**c**) ESO + PF + CR.

Figure 9 presents the results of the SEM experiments at 1000× for three different asphalts. Figure 9a indicates the matrix asphalt, which is nearly homogeneous and exhibits a relatively smooth and flat surface. The irregular lumps in Figure 9b are crumb rubber particles with rough surfaces and more obvious boundaries with the asphalt. This is due to the rough surface of the crumb rubber particles and their poor adhesion to the asphalt, which makes it easy to segregate. It can be seen in Figure 9c that the crumb rubber and asphalt binder adhere to the surface of the polyester fiber, and that the polyester fiber reinforces the asphalt binder as a whole via cross-lap. PF is in contact with the crumb rubber and asphalt at an angle, and no agglomeration occurs. The interfacial behavior in the ESO + PF + CR composite modified asphalt is flat, the boundary is blurred, and the modified materials are uniformly distributed. A comprehensive comparison of Figure 9 shows that the polyester fibers in ESO + PF + CR modified asphalt adsorb the crumb rubber and asphalt and provide reinforcement to the asphalt binder through the cross-lap. The interface is blurred, and the problem of poor storage stability of crumb rubber is improved. The modified material is uniformly distributed in the asphalt binder, forming a solid physical co-mingling.

## 4. Conclusions

This study proved that epoxidized soybean oil and polyester fiber could effectively improve the thermal storage stability of crumb rubber modified asphalt. The synergistic effect and scientific ratio of the three modified materials could improve the high- and low-temperature rheological properties of asphalt. The following conclusions can be obtained:

(1) Polyester fiber’s stabilizing and reinforcing effects mitigate the negative effect of epoxidized soybean oil’s softening effect on the high-temperature rheological properties of modified asphalt. Epoxidized soybean oil can effectively supplement the light components of crumb rubber asphalt to improve the compatibility between crumb rubber and asphalt. The synergistic effects of the CR, PF, and ESO modifiers improve the high- and low-temperature performances of asphalt.
(2) The optimal relative percentage dosages of crumb rubber, polyester fiber, and epoxidized soybean oil with respect to the matrix asphalt quality are determined to be 22%, 0.34%, and 3.21%, respectively, through the response surface method.
(3) Variance analysis and significance analysis of the RSM show that the *p*-values of the model are all less than the significance threshold of 0.05, and that the correlation coefficient and the adjusted determination coefficient are close to 1. The fitting degree is high, and the correlation is significant.
(4) The softening point difference of ESO + PF + CR modified asphalt is 46.9% lower than that of ESO + CR and 51.4% lower than CR. Quantification of the fluorescence area reveals that the stability coefficient of ESO + PF + CR is closest to 1. Polyester fibers have good adhesion with crumb rubber and asphalt, and the overlap between fibers forms a three-dimensional network structure, which plays the role of reinforcement and consolidation in asphalt binder. All these indicate that PF and ESO can improve the storage stability of crumb rubber modified asphalt.

## Figures and Tables

**Table 1:** Technical indexes of polyester fiber.

Material Behavior		Modified Polyesters	
Diameter (μm)	20 ± 5	Extension at break (%)	30 ± 9
Length (mm)	6	Density (g·cm^−3^)	1.36
Acid–alkali resistance	Strong	Melting point (°C)	258
Tensile strength (Mpa)	≥700	Ignition point (°C)	556

**Table 2:** Technical indexes of epoxidized soybean oil.

Testing Item	Enterprise Standard E-10	Test Value
Appearance	Light yellow transparent liquid	Coincidence
Tincture (Platinum–cobalt colorimetric)	≤170	130
Epoxidized value (Hydrochloric acid–acetone)%	≥6.0	6.27
Acid value (mg KOH·g^−1^)	≤0.5	0.4
Iodine number (gI2·(100 g)^−1^)	≤5.0	2.03
Density (20 °C)	0.993 ± 0.005	0.992
Heating loss (125 °C × 2 h)	≤0.2	0.05
Thermal stability (177 °C × 3 h)	≥5.7	6.20
Flash point (Opening cup)	≥280	306

**Table 3:** Technical indexes of matrix asphalt.

Index	Technical Requirement	Test Value
Penetration (25 °C, 100 g, 5 s), 0.1 mm	60~80	71
Penetration index (PI)	−1.5~+1.0	−1.16
Softening point, °C	≥46	47.1
Ductility (10 °C, 5 cm·min^−1^), cm	≥15	76.3
Dynamic viscosity (60 °C), Pas	≥180	197
Flash point (COC), °C	≥260	314
Solubility, %	≥99.5	99.89
Wax content, %	≤2.2	1.5
Density (15 °C), g·cm^−3^	—	1.026

**Table 4:** Dosage of modified materials in each group.

Test Group Number	Epoxidized Soybean Oil	Crumb Rubber	Polyester Fiber
1	4%	18%	0.2%
2	4%	22%	0.2%
3	4%	18%	0.4%
4	4%	22%	0.4%
5	2%	18%	0.3%
6	2%	22%	0.3%
7	6%	18%	0.3%
8	6%	22%	0.3%
9	2%	20%	0.2%
10	2%	20%	0.4%
11	6%	20%	0.2%
12	6%	20%	0.4%
13	4%	20%	0.3%
14	4%	20%	0.3%
15	4%	20%	0.3%
16	4%	20%	0.3%
17	4%	20%	0.3%

**Table 5:** Results of variance analyses of models.

Index	*m*	*S*	*J*_nr0.1_	*J*_nr3.2_
*R* ^2^	0.9643	0.9592	0.9695	0.9875
*Adj.R* ^2^	0.9185	0.9067	0.9303	0.9715
*F*-value	21.03	18.28	24.71	61.53
*p*-value	0.0003	0.0005	0.0002	<0.0001
Lack-of-Fit *p*-value	0.8851	0.6421	0.5066	0.1464
Std. Dev.	0.0049	3.07	1.23	1.47
C.V.%	1.42	2.23	1.63	4.76

**Table 6:** Different modified asphalt upper and lower softening points and differences.

Asphalt Type	Upper Sections	Lower Sections	Differentials
CR	54.5	52.45	1.75
ESO + CR	50.95	49.35	1.6
ESO + PF + CR	54.7	53.85	0.85

**Table 7:** Fluorescence partial area and stability index of different modified asphalts (*I_se_*).

Species	ESO + PF + CR	ESO + CR	CR
Upper sections (*S_t_*)	185.361	401.814	729.18
Lower sections (*S_b_*)	234.442	1118.366	2954.095
Coefficient of stability (*I_se_*)	0.79	0.36	0.25

## Data Availability

Not applicable.
